# The attributable mortality of new-onset acute kidney injury among critically ill patients: a propensity-matched analysis based on a multicentre prospective cohort study

**DOI:** 10.1007/s11255-021-03087-z

**Published:** 2022-01-08

**Authors:** Yi-Jia Jiang, Xiu-Ming Xi, Hui-Miao Jia, Xi Zheng, Mei-Ping Wang, Wen-Xiong Li

**Affiliations:** 1 Department of Surgical Intensive Care Unit, Beijing Chao-yang Hospital, Capital Medical University, 8 Gongren Tiyuchang Nanlu, Chaoyang District, Beijing, 100020 China; 2 Department of Critical Care Medicine, Fuxing Hospital, Capital Medical University, Beijing, China; 3 Department of Epidemiology and Health Statistics, School of Public Health, Capital Medical University, Beijing, China

**Keywords:** Acute kidney injury, New-onset, Attributable, Mortality

## Abstract

**Purpose:**

This study aimed to evaluate the attributable mortality of new-onset acute kidney injury (AKI).

**Methods:**

The data in the present study were derived from a multi-center, prospective cohort study in China that was performed at 18 Chinese ICUs. A propensity-matched analysis was performed between matched patients with and without AKI selected from all eligible patients to estimate the attributable mortality of new-onset AKI.

**Results:**

A total of 2872 critically ill adult patients were eligible. The incidence of new-onset AKI was 29.1% (*n* = 837). After propensity score matching, 788 patients with AKI were matched 1:1 with 788 controls (patients without AKI). Thirty-day mortality was significantly higher among the patients with AKI than among their matched controls (25.5% versus 17.4%, *p* < 0.001). Subgroup analysis in terms of AKI classification showed that there was no significant difference (*p* = 0.509) in 30-day mortality between patients with stage 1 AKI and their matched controls. The attributable mortality values of stage 2 and stage 3 AKI were 12.4% [95% confidence interval (CI) 2.6–21.8%, *p* = 0.013] and 16.1% (95% CI 8.2–23.8%,* p* < 0.001), respectively. The attributable mortality of persistent AKI was 15.7% (95% CI 8.8–22.4%, *p* = 0.001), while no observable difference in 30-day mortality was identified between transient AKI patients and their matched non-AKI controls (*p* = 0.229).

**Conclusion:**

The absolute excess 30-day mortality that is statistically attributable to new-onset AKI is substantial (8.1%) among general ICU patients. However, neither stage 1 AKI nor transient AKI increases 30-day mortality.

**Supplementary Information:**

The online version contains supplementary material available at 10.1007/s11255-021-03087-z.

## Introduction

Acute kidney injury (AKI) is a common complication among critically ill patients and has been increasingly recognized as a risk factor for long-term chronic kidney disease (CKD) development and progression, infection and malignancy [1–3]. The additional mortality attributed to AKI has been researched in previous studies [4–6], but the attributable mortality of new-onset AKI is still unclear among general ICU patients. Attributable mortality would be useful in assessing the number of deaths that might be avoided if new-onset AKI could be prevented [4]. However, it is difficult to evaluate the impact of this disease on mortality because the development of AKI is a multifactorial process and patients with AKI tend to be in worse condition than those without [7]. Therefore, we performed a propensity-matched analysis based on the database of a prospective multi-center cohort study to balance the impact of non-renal variables on outcomes and precisely estimate the mortality attributed to new-onset AKI.

Transient AKI, defined as kidney injury of a duration less than 48 h from AKI onset [8], is usually considered to be “pre-renal dysfunction”, while persistent AKI (kidney injury beyond 48 h) is more closely related to “acute tubular necrosis” [9]. The adverse effect of persistent AKI on prognosis is undisputed; however, whether transient AKI that is quickly resolved will increase mortality is still controversial [10–14]. Thus, according to the duration of renal injury, we conducted a subgroup analysis of matched new-onset AKI patients and non-AKI controls to explore the influence of AKI duration on mortality.

## Methods

The data in this propensity-matched analysis were derived from a prospective multi-center cohort study performed in 18 Chinese ICUs at 16 tertiary hospitals in 7 geographical regions from 1 January 2014 to 31 August 2015 by the China Critical Care Sepsis Trial (CCCST) workgroup. The CCCST was designed to assess the characteristics and outcomes of critically ill patients and consecutively enrolled patients who were admitted to and stayed in the ICU for at least 24 h. For patients who were admitted to the ICU repeatedly during the same hospitalization event, only the first admission was recorded. We excluded patients who developed AKI before admission to the ICU, stayed in the ICU less than 48 h, had chronic kidney disease, had insufficient data, underwent nephrectomy or kidney transplantation, received renal replacement therapy (RRT) for non-renal indications and were < 18 years old.

### Definitions and clinical outcomes

AKI was diagnosed according to the Kidney Disease: improving Global Outcomes (KDIGO) criteria [15]. New-onset AKI was defined as the occurrence of AKI that met the KDIGO criteria within the first 72 h after ICU admission in this study. CKD was defined as an estimated glomerular filtration rate (eGFR) < 60 ml/min/1.73 m^2^ for at least 3 months [16]. Transient AKI was defined as AKI that rapidly reversed within 48 h of onset [8]. Persistent AKI was defined as AKI that lasted beyond 48 h of onset. AKI was classified as persistent if patients met the KDIGO criteria at their last measurement when assessment at 48 h was not possible due to death or discharge from the ICU because treatment was abandoned.

The primary outcome was new-onset AKI within the first 72 h after ICU admission, and the secondary outcome was death within 30 days after ICU admission.

### Data collection

The following information was extracted from the CCCST dataset: demographic characteristics, chronic illnesses (chronic obstructive pulmonary disease or asthma, cardiovascular disease, chronic liver disease, cancer, diabetes, and hypertension), diagnosis, source and type of admission. The severity of illness was indicated by the Acute Physiology and Chronic Health Evaluation (APACHE II) score on the day of ICU admission and the Sequential Organ Failure Assessment (SOFA) score every day thereafter. Baseline serum creatinine, creatinine values every 12 h and hourly urine output at ICU admission and thereafter until transfer out of the ICU, the use of mechanical ventilation, the use of vasopressors (including epinephrine, dopamine, norepinephrine and vasopressin), the use of nephrotoxic drugs (angiotensin-converting enzyme inhibitors, aminoglycosides, and nonsteroidal anti-inflammatory drugs) [17], ICU stay, hospital stay, hospital mortality and 30-day mortality were recorded.

### Statistical analysis

**Fig. 1:**
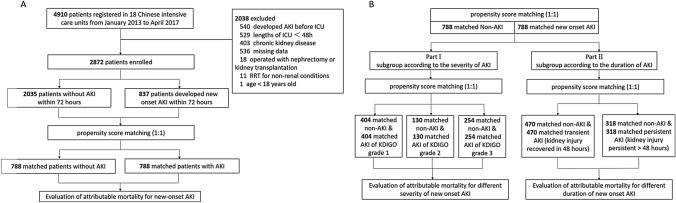
Study flow diagram. **A** Flowchart of all the participants. **B** Flowchart of the subgroup analysis. *AKI* acute kidney injury, *ICU* intensive care unit

Continuous variables are presented as median values (25th and 75th percentiles [interquartile range, IQR]), and categorical variables are presented as percentages. Continuous data between two groups were compared using the Mann–Whitney U test, and categorical variables were compared using the chi square test. We used propensity score matching to evaluate the attributable mortality. The propensity score is the conditional probability that quantifies the likelihood that a study participant is exposed to the exposure factor accounting for the covariates present at baseline. A person in the exposure group is matched with a comparable person who has the most similar propensity scores. Propensity score matching balances the observed covariates between the study groups, similar to a randomized controlled trial. In our study, patients with new-onset AKI were matched to control patients without AKI using propensity scores (Fig. 1A). The propensity score, which was based on baseline characteristics and clinical covariates, was used to adjust the differences between matched patients with and without new-onset AKI and was calculated with logistic regression. The variables age, sex, body mass index (BMI), chronic illnesses, baseline creatinine, non-renal SOFA score, and use of vasopressors, nephrotoxic drugs and mechanical ventilation were included in the estimation of the propensity score. The data of the matched variables in the new-onset AKI cohort were from the day when the most severe AKI staging occurred within the first 72 h after ICU admission. The data of the matched variables in the non-AKI cohort were from the day with the maximum SOFA score within the first 72 h after ICU admission. AKI-exposed patients were matched 1:1 with control patients without AKI according to propensity scores. Then, the 30-day mortality in matched patients with and without new-onset AKI was calculated. The matched total AKI-exposed patients and controls without AKI were sub-grouped by the severity of AKI (KDIGO stage) and the duration (transient and persistent) of new-onset AKI (Fig. 1B). In the propensity score matching analysis, the nearest-neighbor method was applied to create a matched control sample. The caliper width was set to 0.1 of the standard deviation of the logit of the propensity score. The covariate balance before and after matching was examined using standardized differences, with values of 0.15 considered to be evidence of meaningful differences [18]. The attributable mortality of new-onset AKI was calculated by subtracting the mortality of the matched patients without AKI from the mortality of the matched patients with new-onset AKI. The 95% confidence interval (CI) for the attributable mortality difference was calculated by Newcombe’s method [19]. McNemar's test was used for sensitivity analysis to assess the stability of the outcomes [18]. For all analyses, statistical significance was indicated by a two-sided *p* < 0.05. SPSS Statistics 22 (IBM, Chicago, IL) and R 3.6.1 (R Project for Statistical Computing) were used for statistical analyses.

## Results

### Characteristics of the participants and incidence of new-onset AKI

Of the 4910 patients in the cohort, 2872 patients were included in this analysis (Fig. 1A). The median (IQR) age of those patients was 65 (51–77) years. The median (IQR) APACHE score and SOFA score within 24 h after ICU admission were 15 (10–21) and 5 (3–8), respectively. A total of 1909 (66.5%) patients received mechanical ventilation, and 953 (33.2%) patients were diagnosed with sepsis at ICU admission. The median (IQR) length of stay in the hospital was 18 (11–27) days. The unadjusted 30-day mortality rate was 16.4% (*n* = 470) (Table 1).

**Table 1:** Baseline characteristics of patients stratified by new-onset acute kidney injury

	All patients	No AKI	New-onset AKI	*p* value
	*N* = 2872	*N* = 2035	*N* = 837	
Male	1879 (65.4%)	1323 (65.0%)	556 (66.4%)	0.469
Age (years)	65 (51–77)	63 (49–76)	68 (53–80)	< 0.001
BMI (kg/m^2^)	22.5 (19.8–24.5)	22.5 (19.8–24.5)	22.5(19.8–24.5)	0.960
Chronic comorbidities				
COPD/asthma	245 (8.5%)	178 (8.7%)	67 (8.0%)	0.518
Cardiovascular disease	481 (16.7%)	310 (15.2%)	171 (20.4%)	0.001
Hypertension	947 (33.0%)	611 (30.0%)	336 (40.1%)	< 0.001
Diabetes	463 (16.1%)	302 (14.8%)	161 (19.2%)	0.004
Cancer	292 (10.2%)	206 (10.1%)	86 (10.3%)	0.903
Chronic liver disease	86 (3.0%)	50 (2.5%)	36 (4.3%)	0.008
Admission type				
Medical	1073 (37.4%)	711 (34.9%)	362(43.2%)	< 0.001
Surgical	1103 (38.4%)	833 (40.9%)	270 (32.3%)	< 0.001
Emergency	696 (24.2%)	491 (24.1%)	205 (24.5%)	0.8836
APACHEII score	15 (10–21)	14 (9–20)	18 (12–24)	< 0.001
SOFA score	5 (3–8)	5 (3–8)	8 (5–12)	< 0.001
Sepsis	953 (33.2%)	559 (27.5%)	394 (47.1%)	< 0.001
Mechanical ventilation	1909 (66.5%)	1328 (65.3%)	581 (69.4%)	0.032
Use of vasopressors	318 (11.1%)	176 (8.6%)	142 (17.0)	< 0.001
Baseline creatinine (umol/L)	82 (65–95)	81 (65–93)	84 (63–104)	0.091
Use of nephrotoxic drugs	318 (11.1%)	176 (8.6%)	142 (17.0%)	< 0.001
LOS in ICU (days)	6 (3–12)	5 (3–11)	7 (4–14)	< 0.001
LOS in hospital (days)	18 (11–27)	18 (11–27)	18 (11–27)	0.734
Mortality				
ICU mortality	429 (14.9%)	200 (9.8%)	229 (27.4%)	< 0.001
30-day mortality	470 (16.4%)	236 (11.6%)	234 (28.0%)	< 0.001
Hospital mortality	575 (20.0%)	298 (14.6%)	277 (33.1%)	< 0.001

Continuous variables are presented as median and interquartile range

*BMI* body mass index, *COPD* chronic obstructive pulmonary disease, *APACHEII* acute physiologic and chronic health evaluation II, *SOFA* sequential organ failure assessment, *LOS* length of stay

A total of 837 (29.1%) patients developed AKI within the first 3 days after ICU admission, which was classified as new-onset AKI. Furthermore, 421 (50.3%) patients had stage 1 AKI, 133 (15.9%) patients had stage 2 AKI, and 283 (33.8%) patients had stage 3 AKI. A total of 495 (59.1%) patients with AKI recovered renal function within 48 h (transient AKI), and another 342 (40.1%) patients had persistent AKI.

### The attributable mortality of new-onset AKI

**Fig. 2:**
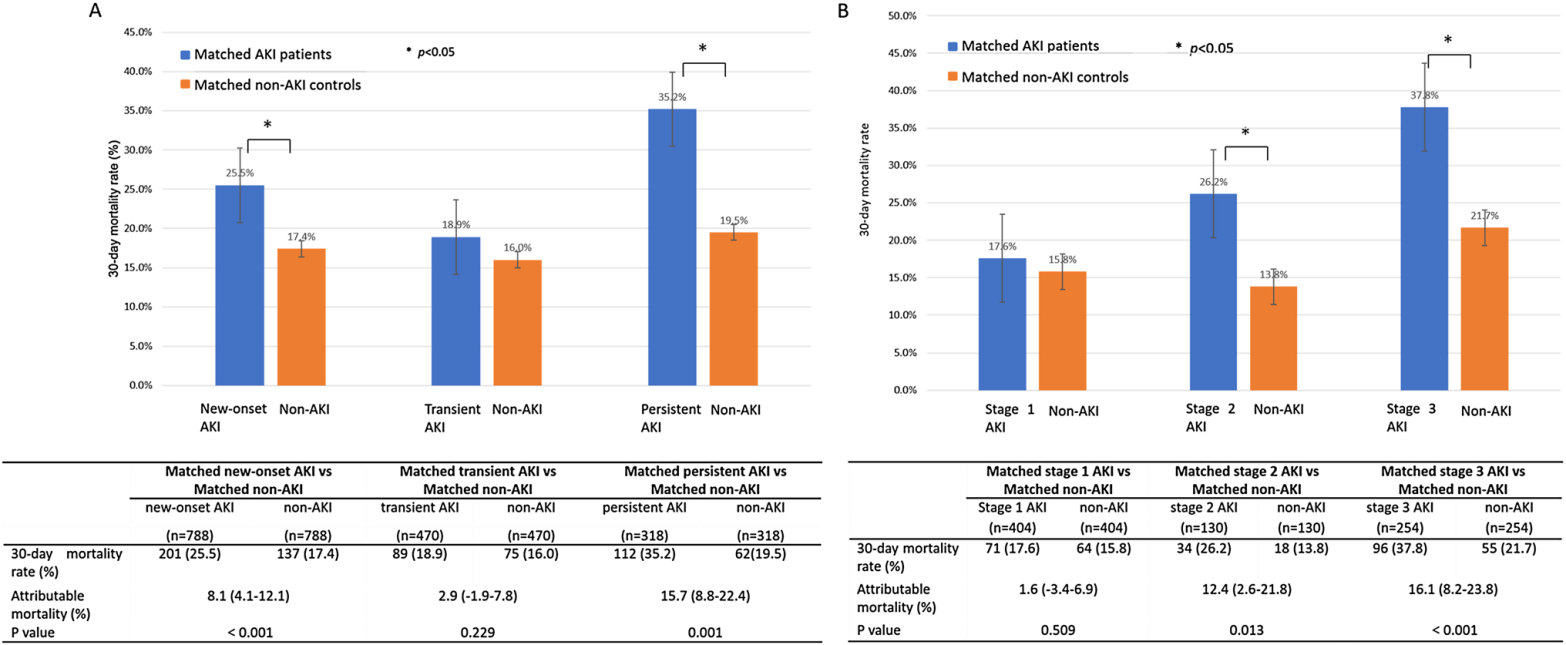
30-day mortality of new-onset AKI patients and matched non-AKI controls. **A** New-onset AKI patients subgrouped according to the duration of kidney injury compared to the controls. **B** New-onset AKI patients subgrouped according to the severity of kidney injury compared to the controls. *AKI* acute kidney injury

A total of 788 patients with new-onset AKI were matched 1:1 with 788 controls without AKI according to propensity scores from all enrolled patients. The 30-day mortality of the matched patients with new-onset AKI was 201 of 788 (25.5%) compared with 137 of 788 (17.4%) for their matched controls without AKI (*p* < 0.001). The attributable mortality of new-onset AKI was 8.1% (95% CI 4.1–12.1%) (Fig. 2A). Patient characteristics of the matched pairs and standardized differences in the covariates after matching are presented in Table 2. The graph of the balance of the propensity scores and the Q–Q plots of the balance of the covariates are shown in Supplemental material Fig. S1 and Supplemental material Fig. S2. Jitter plots of the distribution of propensity scores are shown in Supplemental material Fig. S3.

**Table 2:** Characteristics of a comparison between matched patients with new-onset AKI and their controls without AKI

Variables	New-onset AKI (*n* = 788)	Non-AKI (*n* = 788)	*p* value	Standardized difference (%)
Male	500 (63.5%)	524 (66.5%)	0.21	4.26
Age (years)	66 (53–78)	68 (53–79)	0.14	4.47
BMI (kg/m^2^)	22.5 (19.6–24.2)	22.5 (20.0–24.5)	0.42	1.81
COPD/asthma	73 (9.3%)	66 (8.4%)	0.53	− 0.93
Cardiovascular disease	143 (18.1%)	154 (19.5%)	0.48	3.75
Hypertension	291 (36.9%)	305 (51.2%)	0.47	4.11
Diabetes	131 (16.6%)	148 (18.8%)	0.26	− 0.32
Cancer	69 (8.8%)	80 (10.2%)	0.34	1.66
Chronic liver disease	30 (3.8%)	31 (3.9%)	0.89	− 1.24
Non-renal SOFA score	6 (4–9)	6 (4–9)	0.12	2.55
Sepsis	327 (41.5%)	353 (44.8%)	0.19	2.52
Baseline creatinine (μmol/L)	85 (66–98)	83 (62–100)	0.89	0.05
Use of nephrotoxic drugs	113 (14.7%)	129 (16.4%)	0.26	− 1.68
Mechanical ventilation	541 (68.7%)	544 (69.0%)	0.87	− 1.37
Use of vasopressors	260 (33.0%)	291 (36.9%)	0.10	1.03

Values are median (interquartile range) or *n* (%)

*BMI* body mass index, *SOFA* sequential organ failure assessment, *COPD* chronic obstructive pulmonary disease

### Subgroup analysis of the effects of different severity levels and durations of AKI on attributable mortality

Matched AKI-exposed patients and patients without AKI were sub-grouped according to the severity (KDIGO grades 1–3) and duration (transient and persistent) of kidney injury (Fig. 1B). A total of 404 patients with stage 1 AKI, 130 patients with stage 2 AKI and 254 patients with stage 3 AKI were matched 1:1 with controls without AKI. The attributable mortality of AKI differed according to the severity level (Fig. 2B). The 30-day mortality of matched patients with AKI grade 1 and the matched controls was not significantly different (*p* = 0.509). The attributable mortality values of stage 2 AKI and stage 3 AKI were 12.4% (95% CI 2.6–21.8%, *p* = 0.013) and 16.1% (95% CI 8.2–23.8%,* p* < 0.001), respectively. A total of 470 patients with transient AKI and 318 patients with persistent AKI were matched 1:1 with their respective controls without AKI. The attributable mortality of persistent AKI was 15.7% (95% CI 8.8–22.4%, *p* = 0.001), while the mortality of patients with transient AKI and their matched non-AKI controls showed no significant difference (*p* = 0.229) (Fig. 2A).

To assess the relationship between AKI stage and duration, we calculated the mortality of each group in accordance with AKI stage and duration (Supplemental material Fig. S4). Among the 470 patients with transient AKI, most of them (349, 74.3%) had stage 1 AKI, 54 (11.5%) had stage 2 AKI, and 67 (14.3%) had stage 3 AKI; none of the groups showed differences in 30-day death compared to the corresponding matched non-AKI patients. Among those with persistent AKI, more than half of them (187, 58.8%) had stage 3 AKI. Compared to matched non-AKI patients, those with stage 2 and stage 3 persistent AKI had a significantly higher 30-day death rate, with the attributable mortality of persistent stage 2 AKI and persistent stage 3 AKI being 17.1% (95% CI 4.0–29.6%, *p* = 0.011) and 18.2% (95% CI 8.8–27.1%, *p* < 0.001), respectively. However, there was no difference in 30-day mortality between the matched patients with persistent stage 1 and non-AKI controls (25.5% versus 20.0%,* p* = 0.459).

### Sensitivity analysis

Propensity score analysis relies on the assumption that all important covariates have been measured, and thus, any bias due to unmeasured covariates can be ignorable. We conducted a sensitivity analysis to assess the observed factors with the “possible” presence of hidden bias from unmeasured covariates. The hidden bias after matching was measured by two odds (gamma) tests. These two different odds are defined as the odds of a treated person receiving the treatment and the odds for a comparison person receiving the treatment [18]. The output, presented in Supplemental material Table S1, suggested that the statistical significance would not change even if the new-onset AKI group was as much as three times more likely to suffer AKI than their matched comparison group, with the upper bound of the p value less than 0.001 at gamma = 3. In other words, the new-onset AKI estimate for 30-day mortality is robust to hidden bias from unmeasured covariates up to a gamma coefficient of 3.

## Discussion

In this propensity-matched analysis, we found that the estimated absolute excess 30-day mortality statistically attributable to new-onset AKI was 8.1% (95% CI 4.1–12.1%). This finding further strengthens the role of AKI as an independent risk factor for mortality and is useful in calculating the sample size for future clinical research on AKI in critically ill patients. To the best of our knowledge, this is the first study to describe the magnitude of the excess mortality attributable to new-onset AKI in critically ill patients. Few previous studies have evaluated the mortality attributable to kidney injury. A study by Vaara ST and colleagues estimated that the absolute excess 90-day mortality statistically attributed to acute kidney injury was 8.6% among general ICU patients [4], which is similar to our result for the 30-day attributable mortality of new-onset AKI. However, in a study by Levy EM and colleagues, the mortality attributable to renal failure was 27% (*P* < 0.001) in patients undergoing radiocontrast procedures [5], and in a study by Cheyron D and colleagues, severe acute renal failure was associated with a higher mortality of 51% in critically ill patients with cirrhosis [6]. The attributable mortality in these two studies was higher than our result, and the variation may be due to the following reasons: these two studies paired ICU admission diagnosis and APACHE II severity score [6] or age and baseline creatinine [5]. To this basis, we added sex, BMI, chronic illnesses, non-renal SOFA score, and the use of vasopressors, nephrotoxic drugs and mechanical ventilation to the estimation of the propensity score to better balance the non-renal variables in the new-onset AKI group and non-AKI group. Moreover, both of these studies used acute renal failure as the diagnostic criteria, reflecting that patients with kidney injury were in more serious condition, which may also lead to differences in mortality rates. Since it is almost impossible to confirm the onset time of AKI in patients who developed AKI before ICU admission and the SOFA score at that time, we included only new-onset AKI patients, so our result may be closer to the impact of AKI itself on mortality.

On the basis of matching patients with new-onset AKI of any severity to those without AKI, we further evaluated the impact of different stages and durations of AKI on mortality. Patients with KDIGO stage 1 AKI did not demonstrate substantially higher mortality than their matched non-AKI controls. This finding may signify that stage 1 AKI itself does not expose patients to worse outcomes when comorbidities and disease severity are taken into consideration. Stage 2 and stage 3 AKI significantly increased 30-day mortality, and the magnitude of statistically attributable mortality increased with the severity of AKI. Few available previous studies support this increasing trend [4, 6]. Similar results were also found in the subgroup analysis in terms of AKI duration. Transient AKI, defined as renal function recovery within 48 h after AKI onset, is a rapidly reversible form of AKI and has been recognized as a benign form [12]. Patients with transient AKI had a lower risk of death than patients with persistent AKI [12, 13] and had mild long-term adverse outcomes [10, 11]. To precisely calculate the duration of kidney injury, we excluded patients who had AKI before admission and enrolled only new-onset AKI patients in this analysis. Our study confirmed that transient AKI itself did not increase the risk of death, regardless of AKI stage. This finding corroborates the previous understanding of transient AKI; that is, transient AKI manifests only as a reversible loss of renal function without any detectable structural kidney damage [20]. To our knowledge, this is the first study to clarify the effect of transient AKI on short-term prognosis in a heterogeneous population of critically ill patients.

Our results showed that stage 2 and stage 3 AKI increased mortality only if the kidney injury lasted more than 48 h. We could consider stage 1 AKI and transient AKI to be mild AKI and persistent stage 2 and persistent stage 3 AKI to be severe AKI according to whether AKI itself increased mortality. This classification method combining the AKI stage and duration may help clinicians better predict prognosis.

This study still has a few limitations. First, the follow-up period was relatively short. Transient AKI and stage 1 AKI had no additional contribution to the 30-day mortality in our study but may have an impact on the long-term prognosis, and future research should continue to explore the long-term mortality attributable to AKI. Second, we defined AKI that appeared in the first 3 days after admission as new-onset AKI and may have ignored AKI that developed afterward. However, previous studies found that approximately 93.1% of AKI cases involving critically ill patients occurred within the first 72 h after admission to the ICU [4, 21, 22], and 87.0% of AKI patients developed the condition the first 3 days in our study, so our findings are generalizable, as our study population is representative of the vast majority of AKI patients. Third, due to the smaller sample size, the separately calculated attributable mortality rates for different stages and durations of new-onset AKI have relatively wide CIs, and the findings should be interpreted with caution. Fourth, we did not include the variable of diagnosis leading to ICU admission in the propensity score analysis because multi-categorical variables cannot be included in the model. However, we analyzed the influence of the unmeasured variables on the stability of the propensity score analysis result, and the sensitivity analysis showed that the variables not included in the analysis were not sufficient to influence the stability of the result.

## Conclusion

In this propensity score-matched analysis, we estimated that the attributable mortality of new-onset AKI was 8.1% (95% CI 4.1–12.1%) among general ICU patients. Transient AKI and stage 1 AKI themselves did not increase mortality, and the attributable mortality rates of persistent stage 2 and persistent stage 3 AKI were 17.1% (95% CI 4.0–29.6%) and 18.2% (95% CI 8.8–27.1%), respectively.

## Supplementary Information

Below is the link to the electronic supplementary material.

Supplementary file1 (DOCX 1727 kb)

## Data Availability

The datasets used and/or analyzed during the current study are available from the corresponding author on reasonable request.
