# Supplementary material for: The attributable mortality of new-onset acute kidney injury among critically ill patients: a propensity-matched analysis based on a multicentre prospective cohort study
Source: Int Urol Nephrol. 2022 Jan 8;54(8):1987–94. doi: 10.1007/s11255-021-03087-z (PMC9262803; doi:10.1007/s11255-021-03087-z)
Supplement: Supplementary file 1 — Supplementary file1 (DOCX 1727 kb) [file 11255_2021_3087_MOESM1_ESM.docx]

**Data Supplement**

**
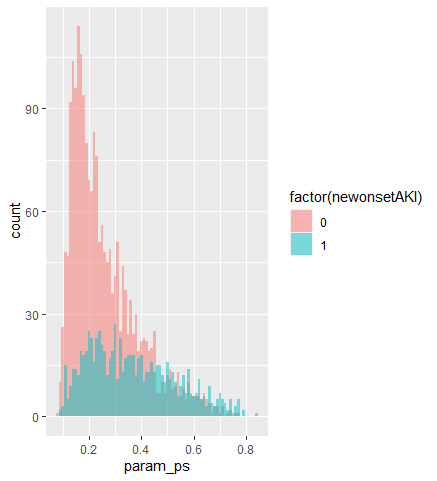
**

Fig. S1 Balance of the propensity score. In this figure, a substantial overlap (“common support”) is observed over the range of the propensity score between the new-onset AKI group and non-AKI group, suggesting a large area of common support among the patients. A large area of common support increases confidence that the observed effect would generalize to the entire population being represented by the sample. On the other hand, a small area of common support indicates that the observed effect would be valid only for a small subgroup of the population.


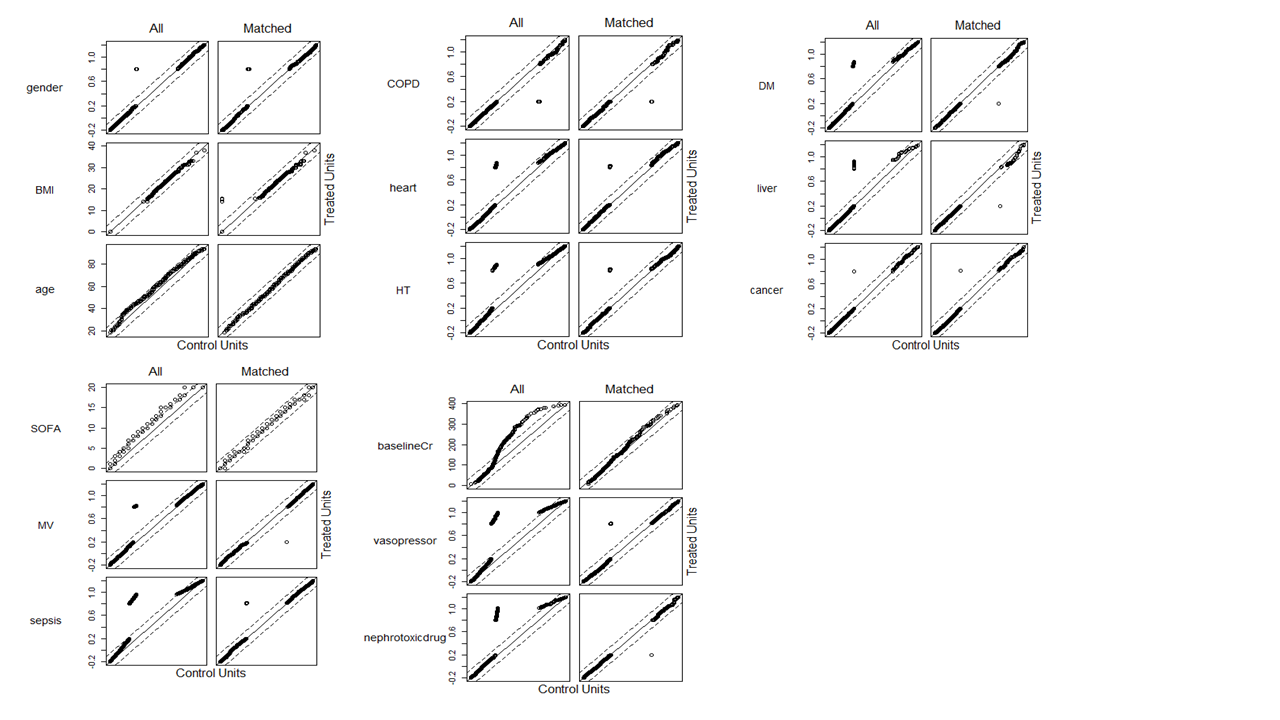


Fig S2 Balance of the covariates. Balance was also examined graphically for the covariates after matching. The values of a covariate in the new-onset AKI group are plotted against those in the non-AKI group in a quantile-quantile (Q-Q) plot. If the distributions lie along a 45-degree line, the covariate is deemed to be balanced.


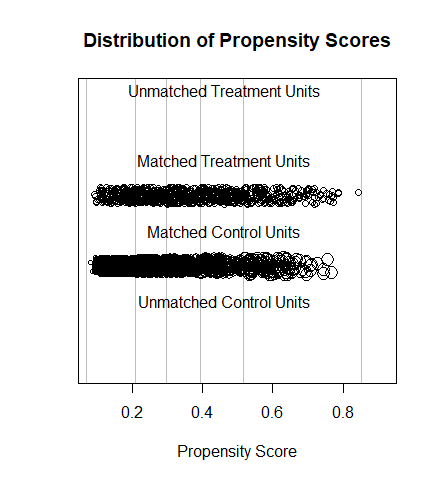


Fig S3 Jitter plots of the distribution of propensity scores. Each circle represents a patient’s propensity score, and the size of the circle corresponds to his or her weight. The absence of circles in the “unmatched treatment units” and “unmatched control units” indicates that there are no unmatched cases.

**
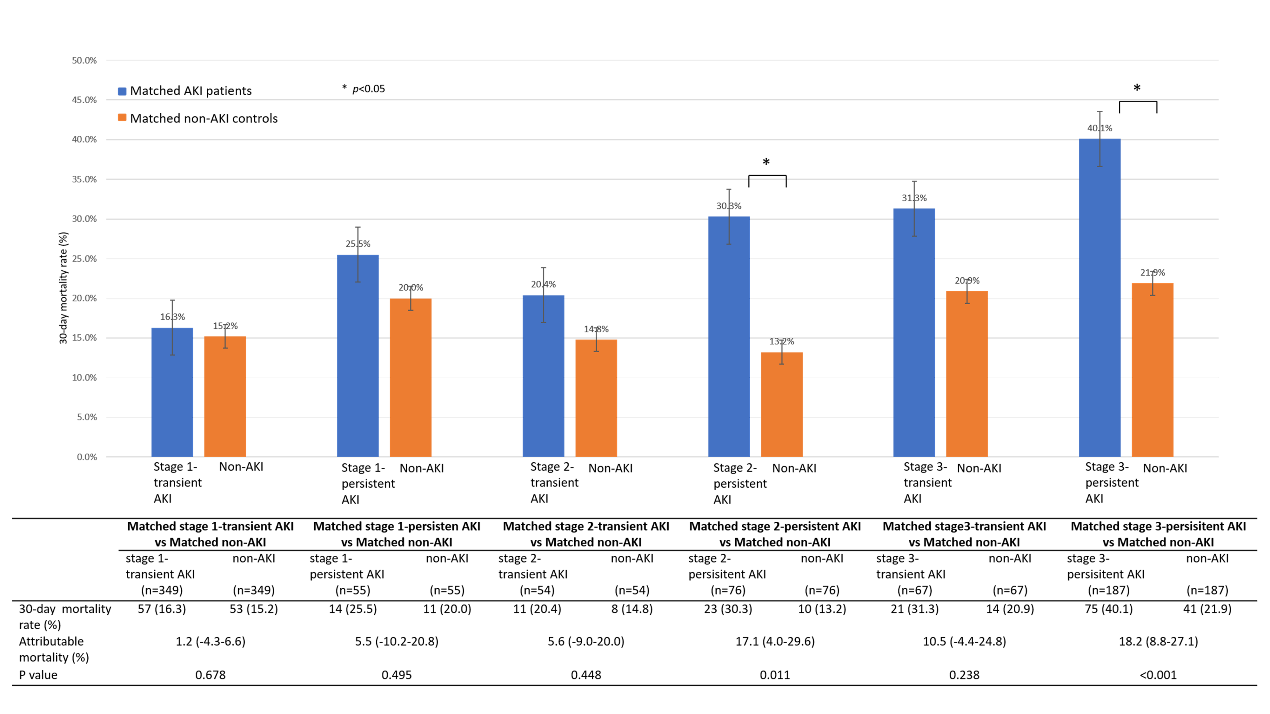
**

Fig S4. Thirty-day mortality rates of new-onset AKI patients according to both duration and severity of kidney injury compared to their matched controls. This figure shows the mortality of each group according to AKI stage and duration, accounting for the relationship between AKI stage and duration. There was no difference in 30-day death rate between patients with transient AKI and the matched non-AKI patients. For persistent AKI, more than half of the patients had stage 3 AKI. Compared to the matched non-AKI patients, those with persistent stage 2 and stage 3 AKI had a significantly higher rate of 30-day death, with the attributable mortality values of persistent stage 2 AKI and persistent stage 3 AKI being 17.1% (95% CI 4.0-29.6%, p = 0.011) and 18.2% (95% CI 8.8-27.1%, p < 0.001), respectively.

**Table S1.** Results of the sensitivity analysis of 30-day mortality

| Gamma | Lower bound | Upper bound |
| --- | --- | --- |
| 1 | 0 | 0.00000 |
| 2 | 0 | 0.00000 |
| 3 | 0 | 0.00005 |

The gamma coefficient represents the odds of differential assignment to new-onset AKI due to unobserved factors.

Propensity score analysis relies on the assumption that all important covariates have been measured and thus that any bias due to unmeasured covariates can be ignorable. We conducted a sensitivity analysis by assessing how sensitive the observed (estimated) effect is to the “possible” presence of (hidden) bias from unmeasured covariates. Gamma, the odds of differential assignment to new-onset AKI due to unmeasured factors, represents the bias after matching. For instance, if gamma=1, a patient with new-onset AKI and their matched control in every match would have exactly the same chance of exposure to AKI, i.e., no bias. Table S1 suggests that the propensity score analysis was robust even if the patients in the new-onset AKI group were as much as three times more likely to have AKI than their matched cases, with the upper bound of the p-value less than 0.001 at gamma = 3.
